# Mutant desmin substantially perturbs mitochondrial morphology, function and maintenance in skeletal muscle tissue

**DOI:** 10.1007/s00401-016-1592-7

**Published:** 2016-07-08

**Authors:** Lilli Winter, Ilka Wittig, Viktoriya Peeva, Britta Eggers, Juliana Heidler, Frederic Chevessier, Rudolf A. Kley, Katalin Barkovits, Valentina Strecker, Carolin Berwanger, Harald Herrmann, Katrin Marcus, Cornelia Kornblum, Wolfram S. Kunz, Rolf Schröder, Christoph S. Clemen

**Affiliations:** 1 Institute of Neuropathology, University Hospital Erlangen, Schwabachanlage 6, 91054 Erlangen, Germany; 2 Functional Proteomics, SFB815 Core Unit, Medical School, Goethe University, 60590 Frankfurt, Germany; 3 German Center for Cardiovascular Research (DZHK), Partner site RheinMain, Frankfurt am Main, Germany; 4 Life and Brain Center, University Hospital of Bonn, 53127 Bonn, Germany; 5 Department of Epileptology, University Hospital of Bonn, 53105 Bonn, Germany; 6 Medizinisches Proteom-Center, Medical Faculty, Ruhr University Bochum, 44801 Bochum, Germany; 7 Department of Neurology, University Hospital Bergmannsheil, Ruhr-University Bochum, 44789 Bochum, Germany; 8 Center for Biochemistry, Institute of Biochemistry I, Medical Faculty, University of Cologne, Joseph-Stelzmann-Str. 52, 50931 Cologne, Germany; 9 Department of Neurology, University Hospital of Bonn, 53105 Bonn, Germany; 10 Center for Rare Diseases Bonn (ZSEB), University Hospital of Bonn, 53105 Bonn, Germany

**Keywords:** Desminopathy, R350P desmin, R349P desmin knock-in, Desmin knock-out, Intermediate filament, Myofibrillar myopathy, Protein aggregate myopathy, Mitochondria, Proteome, mtDNA

## Abstract

Secondary mitochondrial dysfunction is a feature in a wide variety of human protein aggregate diseases caused by mutations in different proteins, both in the central nervous system and in striated muscle. The functional relationship between the expression of a mutated protein and mitochondrial dysfunction is largely unknown. In particular, the mechanism how this dysfunction drives the disease process is still elusive. To address this issue for protein aggregate myopathies, we performed a comprehensive, multi-level analysis of mitochondrial pathology in skeletal muscles of human patients with mutations in the intermediate filament protein desmin and in muscles of hetero- and homozygous knock-in mice carrying the R349P desmin mutation. We demonstrate that the expression of mutant desmin causes disruption of the extrasarcomeric desmin cytoskeleton and extensive mitochondrial abnormalities regarding subcellular distribution, number and shape. At the molecular level, we uncovered changes in the abundancy and assembly of the respiratory chain complexes and supercomplexes. In addition, we revealed a marked reduction of mtDNA- and nuclear DNA-encoded mitochondrial proteins in parallel with large-scale deletions in mtDNA and reduced mtDNA copy numbers. Hence, our data demonstrate that the expression of mutant desmin causes multi-level damage of mitochondria already in early stages of desminopathies.

**Electronic supplementary material:**

The online version of this article (doi:10.1007/s00401-016-1592-7) contains supplementary material, which is available to authorized users.

## Introduction

The term “desminopathies” stands for a group of familial and sporadic myopathies and cardiomyopathies [15] that are caused by mutations in the human desmin (*DES*) gene on chromosome 2q35, which encodes the muscle-specific intermediate filament protein desmin [42]. Desminopathies belong to the expanding group of protein aggregate myopathies (PAMs) and are currently classified as a subgroup of myofibrillar myopathies (MFMs) [66]. The latter comprise a genetically and clinically heterogeneous group of striated muscle diseases that share the morphological characteristics of desmin-positive protein aggregates and degenerative changes of the myofibrillar apparatus. MFMs are caused by mutations in genes coding for structural components of the myofibrillar apparatus (filamin-C, myotilin, ZASP, FHL1, titin), the extrasarcomeric cytoskeleton (desmin, plectin), and components with functions in protein quality control (DNAJB6, BAG-3, αB-crystallin) [66].

Since the first description of human desmin mutations in 1998 [29], more than 70 myopathy- or cardiomyopathy-causing desmin mutations have been reported [15]. The vast majority of desminopathies follows an autosomal-dominant trait of inheritance. The few autosomal-recessive cases may be subdivided in cases with maintained expression of mutant desmin [2, 9, 29, 56, 60] and others with a complete lack of desmin [7, 23, 31, 51]. While the former resulted in myopathies and cardiomyopathies with desmin-positive protein aggregates, the latter were not reported to display aggregation pathology. Human desminopathies are clinically highly variable with disease onsets ranging from the first to the eighth decade of life and may manifest with either pure skeletal muscle or cardiac disease symptoms, or with a combination of both [15]. To date, curative treatment is neither available for desminopathies nor other forms of PAMs.

How do human desmin mutations inflict progressive striated muscle damage? The intermediate filament protein desmin is the major component of the three-dimensional extrasarcomeric cytoskeleton and exerts multiple roles in the alignment and anchorage of myofibrils, the positioning of mitochondria and myonuclei, mechanosensing, stress endurance, and cell signaling [15]. Since muscle biopsies from desminopathy patients represent only late stages of the disease and are only available in small amounts, patient-mimicking disease models are required to decipher the sequential steps of the in vivo molecular pathogenesis. We recently reported the generation and characterization of R349P (c.1045_1047delAGG>insCCC) desmin knock-in mice, which harbor the orthologue of the most frequently occurring human desmin missense mutation R350P (c.1049G>C) [16]. These mice display age-dependent skeletal muscle weakness, dilated cardiomyopathy, and cardiac arrhythmias and conduction defects. On the molecular level, the point-mutated desmin led to aberrant subcellular localization and increased turnover of desmin and its binding partners. We further demonstrated that the progressive muscle pathology is primarily caused by disruption of the extrasarcomeric intermediate filament network rather than by the presence of pathological protein aggregates [16].

Several previous studies on muscle biopsy specimens from human desminopathies and other forms of MFMs reported on mitochondrial pathology [15, 24, 33, 34, 50, 67, 68]. Moreover, various mitochondrial abnormalities were described in a transgenic mouse model expressing p.L345P mutant desmin [38] and in desmin knock-out mice [15, 40, 41, 44, 54, 55, 73]. In this study, we investigated the relationship between mutant desmin and mitochondrial pathology by comprehensive and multi-level analyses in human and murine desminopathies.

## Materials and methods

### R349P desmin knock-in and desmin knock-out mice

In this study we used the following desminopathy mouse models: hetero- and homozygous R349P (c.1045_1047delAGG>insCCC) desmin knock-in mice B6J.129Sv-*Des*^tm1.1Ccrs^ (http://www.informatics.jax.org/allele/MGI:5708562; synonym: B6J.129Sv-*Des*^tm1(R349P)Cscl&Rfsr^) [16] as well as desmin knock-out mice B6J.129S2/Sv-*Des*^tm1Cba^ (http://www.informatics.jax.org/allele/MGI:2159584) [40]. Breeding pairs of the latter were received by courtesy from Denise Paulin, Université Pierre et Marie Curie, Paris, France. The mice were handled in accordance with the German Animal Welfare Act (Tierschutzgesetz) as well as the German Regulation for the protection of animals used for experimental purposes or other scientific purposes (Tierschutz-Versuchstierverordnung), and the investigations were approved by the responsible governmental animal care and use office [Landesamt für Natur, Umwelt und Verbraucherschutz North Rhine-Westphalia (LANUV NRW), Recklinghausen, Germany; reference numbers 84-02.04.2014.A262 and 84-02.05.40.14.057].

### Human skeletal muscle biopsy material

Tissue samples derived from diagnostic vastus lateralis muscle biopsies of two patients from a previously reported family with a heterozygous c.1049G>C (R350P) desmin mutation [3] and of one previously reported patient harboring the heterozygous c.735G>C desmin mutation leading to the expression of two mutant desmin protein species (E245D and D214_E245del) [14] were obtained from the Department of Neurology, University Hospital Bergmannsheil, Ruhr-University, Bochum, Germany and the Department of Neurology, University Hospital Bonn, Germany, respectively. Human desmin reference sequences: NM_001927.3; P17661.

### Antibodies

Both wild-type and R350P/R349P mutant desmin proteins were detected by two commercially available desmin antibodies [mouse monoclonal antibody (mAb), D1033, Sigma-Aldrich (St. Louis, MO, USA), 1:1000 in TBS-T for western blotting; rabbit polyclonal antibodies (pAb), #10570, Progen Biotechnik GmbH (Heidelberg, Germany), 1:100 in PBS for immunofluorescence], GAPDH by a mouse mAb [G9295, Sigma-Aldrich (St. Louis, MO, USA), 1:10,000 in TBS-T for western blotting], multiple complex I subunits by a rabbit anti-serum ([8], antibody #55, purified bovine complex I was used for immunization, 1:10,000 in TBS-T for western blotting), complex II (SDHA) by a mouse mAb (#459200, Invitrogen, 1:1000 in TBS-T for western blotting), complex III [UQCRC2 subunit (Core protein II)] by a mouse mAb (A11143, Molecular Probes, 1:3000 in TBS-T for western blotting), complex IV (CoxVIa subunit) by a rabbit anti-serum ([30], antibody #90, 1:10,000 in TBS-T for western blotting), complex IV (CoxI subunit) by a mouse mAb (#59600, Invitrogen, 1:100 in PBS for immunofluorescence), complex V (ATP5A subunit) by a mouse mAb (#459240, Invitrogen, 1:1000 in TBS-T for western blotting), and multiple subunits of complexes I–V by a ready-to-use mixture of mouse mAbs (MS604, MitoSciences, 1:5000 in TBS-T for western blotting).

### Preparation of skeletal muscle cryosections, histochemistry, and immunofluorescence stains

Skeletal muscle specimens were collected and immediately frozen in liquid nitrogen-cooled isopentane. Cryostat sections of 5 µm thickness were placed on microscope slides and air-dried for 30 min. Histochemistry was performed using routine staining protocols [22], and images were captured using an Olympus CX41 light microscope (Olympus, Hamburg, Germany). For immunofluorescence analyses, cryosections were fixed for 5 min with acetone, air-dried for 30 min, and permeabilized with PBS containing 0.2 % Triton X-100 for 15 min. Non-specific binding was blocked with 10 % fetal calf serum, 1 % goat serum and 0.1 % sodium azide in PBS for 1 h at room temperature. Incubation with primary antibodies diluted in PBS with 3 % BSA was done overnight at 4 °C or for 1 h at room temperature. After washing, sections were incubated with appropriate goat anti-mouse or anti-rabbit Alexa Fluor 488, 633, and 647 antibodies (1:200, Molecular Probes/Life Technologies GmbH, Darmstadt, Germany), and finally washed with PBS and mounted in Mowiol for analysis using a Leica TCS SP5/AOBS/tandem scanning system (Leica Microsystems GmbH, Wetzlar, Germany) with emission detection in sequential mode equipped with the Leica LAS-AF software (v. 2.7.3.9723).

### Preparation of isolated skeletal muscle fibers and immunofluorescence analysis

Preparation of single soleus muscle fibers was performed as described [64]. Mice were anesthetized with isoflurane (Abbot) and heart-perfused with 2 % paraformaldehyde (PFA) in PBS by cutting off the right and punctuating the left heart ventricle. Subsequently, soleus muscles were dissected and fixed for 15 min in 2 % PFA in PBS, followed by washing with PBS. After removing remaining tendons, muscles were cut into two halves and divided into 5–7 portions, depending on the size of the muscle. The bundles of muscle were teased into single fibers using 90° angled, fine-tipped needles (Carl Reiner GmbH, Vienna, Austria), mounted on coated glass slides (Superfrost Plus, Thermo Scientific), air-dried and stored at −80 °C.

For immunofluorescence analyses, isolated fibers were thawed at room temperature for 15 min, encircled with a hydrophobic pen, and permeabilized in 0.1 % Triton X-100 in PBS for 30 min. To reduce background signal caused by endogenous mouse immunoglobulins, the Mouse on Mouse (M.O.M.) Basic Kit (Vector Laboratories) was used according to the manufacturer’s instructions. 200 μl of “M.O.M. Diluent” were applied for 5 min, followed by 1 h incubation with primary antibodies (mouse monoclonal anti-CoxI subunit and rabbit polyclonal anti-desmin) diluted in “M.O.M. Diluent”. After 10 min incubation with 200 μl “M.O.M. Biotinylated Anti-Mouse IgG Reagent” diluted 1:250 in “M.O.M. Diluent” per slide, slides were washed twice and secondary antibodies-dilution, which contained Streptavidin Protein-DyLight 550 conjugate (Thermo Fischer Scientific) and goat anti-rabbit Alexa Fluor 488 (Life Technologies), was added for 1 h at room temperature. After a short washing with PBS, samples were air-dried and mounted in Mowiol. Images were recorded using a LSM780 fluorescence laser scanning microscope (Carl Zeiss, Jena, Germany) equipped with a Plan-Apochromat 63 × 1.4NA objective lens. Images were obtained using the LSM780 module and the Zeiss ZEN software.

### Ultrastructural analysis

For transmission electron microscopy, skeletal muscle specimens were fixed in freshly prepared 4 % formaldehyde, 15 % saturated picric acid and 0.5 % glutaraldehyde in 0.1 M phosphate buffer pH 7.4 overnight at 4 °C, postfixed in 0.5 % osmium tetroxide, washed and counterstained with uranyl acetate, dehydrated in graded ethanol concentrations, and embedded in epoxy resin. Ultra-thin sections were prepared (Ultracut S; Leica, Germany) and examined with a LEO 906E or LEO 910 transmission electron microscope (Carl Zeiss GmbH, Oberkochen, Germany).

### Citrate synthase activity

Snap-frozen soleus muscles were pulverized in a mortar in liquid nitrogen and small amounts were dissolved in 300 µl PBS, sonicated in short time-intervals on ice, and centrifuged at 16,000×*g* for 5 min at 4 °C. Protein concentration was determined using a fluorometric dye (ProStain, Active Motif, Carlsbad, CA, USA) as well as densitometry analysis of Coomassie Brilliant Blue stained SDS gels both before and after adjustment of a total protein concentration of 0.4 mg/ml.

Citrate synthase activity was determined following the reduction of 5,5′-dithiobis-(2-nitrobenzoic acid) (DTNB) by CoA-SH liberated by the citrate synthase reaction in the presence of oxaloacetate and acetyl-CoA as described previously [39, 76]. For this assay, samples were diluted 1:2 to 1:6 in 0.1 M Tris-HCl pH 7.0 and 80 µl samples were added to 920 µl incubation mix [720 µl H_2_O, 100 µl 0.1 mM DNTB (Sigma, D8130; in 1 M Tris-HCl pH 8.1), 25 µl 10 % Triton X-100, 50 µl 10 mM oxalacetate (Sigma, O-4126; in 0.1 M triethanolamine-HCl pH 8.0 with 5 mM EDTA), 25 µl 12.2 mM acetyl-CoA (Sigma, A-2181)] in a plastic cuvette. The linear absorbance at 412 nm was monitored for 200 s in a U-2000 spectrophotometer (Hitachi).

### Respiratory complex I and IV activities

Samples of snap-frozen muscle tissue were homogenized in 0.1 M phosphate buffer pH 7.4 (25 mg tissue per 1 ml buffer) three times for 15 s at 24,000 rpm with an Ultra-Turrax homogenizer (IKA, Staufen, Germany) and centrifuged at 16,000×*g* for 15 min at 4 °C. The supernatants contained all cytosolic (99–100 % of lactic dehydrogenase; six independent control biopsy samples) and all mitochondrial matrix enzymes (90–95 % of citrate synthase; six independent control biopsy samples). The pellets, which contained all mitochondrial inner membrane associated enzyme activities (99–100 % of cytochrome *c* oxidase and NADH:CoQ1 reductase; six independent control biopsy samples), were re-suspended in half of the volume of the initially added phosphate buffer. Supernatants were kept snap-frozen in liquid nitrogen until use; pellet fractions were immediately used for the measurements.

The activity of rotenone-sensitive NADH:CoQ 1 oxidoreductase (complex I) was measured at 30 °C using a dual-wavelength spectrophotometer (Aminco DW 2000, SLM Instruments, Rochester, NY, USA) at 340/380 nm (*ε*_red-ox_ = 5.5 mM^−1^ cm^−1^). The reaction medium contained 50 mM KCl, 1 mM EDTA, 10 mM Tris-HCl pH 7.4, 1 mM KCN, 100 µM CoQ_1_, and 150 µM NADH. The assay was initiated by addition of the sample and the velocity of NADH oxidation was monitored. To determine the rotenone-insensitive NADH oxidation rate, 20 µM rotenone was added to the assay mixture after 2 min. The shown activities are differences between the total NADH oxidation rate and the rotenone-insensitive NADH oxidation rate. The cytochrome *c* oxidase (complex IV) activities were measured at 30 °C in 0.1 M potassium phosphate buffer (pH 7.4) containing 0.02 % laurylmaltoside (Sigma, Munich, Germany) monitoring the oxidation of ferrocytochrome c in its β-band at the wavelength pair 510/535 nm (*ε*_red-ox_ = 5.9 mM^−1^ cm^−1^). To obtain reduced cytochrome c, oxidized bovine heart cytochrome *c* (purity 99 %, Sigma, Munich, Germany) was reduced with ascorbate, desalted on a Sephadex-G25 column, and stored in liquid nitrogen until use.

### SDS-PAGE of samples from skeletal muscle tissue

For quantitative immunoblotting extraction of proteins from skeletal muscle tissue was done according to [13]. Small amounts of snap-frozen tissue were pulverized in a mortar in the presence of liquid nitrogen, taken up in lysis buffer (5 mM Tris-HCl pH 6.8, 10 % SDS, 0.2 M DTT, 1 mM EDTA) and heated at 95 °C for 5 min. Subsequently, the lysates were sonicated six times for 10 s, again heated for 5 min at 95 °C, and centrifuged at 16,000×*g* for 5 min. Protein concentrations of the supernatants were determined using a fluorometric dye (ProStain, Active Motif, Carlsbad, CA, USA), adjusted to 3 mg/ml by addition of lysis buffer, again quantitated, and finally adjusted to 1 mg/ml by addition of 1× SDS sample buffer (25 mM Tris-HCl pH 6.8, 0.8 % SDS, 2 % 2-mercaptoethanol, 4 % glycerol, 0.001 % bromophenol blue). Samples were boiled once more before application to a SDS-polyacrylamide gel electrophoresis and run under standard conditions. For detection of proteins, both Coomassie Brilliant Blue staining and immunoblotting employing appropriate antibodies were used.

### Quantitative mass spectrometric analyses of soleus muscle lysates

Soleus muscle lysates adjusted to 1 mg/ml total protein in SDS sample buffer (prepared as described above) were used for label-free quantitative mass spectrometric analyses. For fractionation, 20 µg of protein per lane were loaded onto 4–12 % Bis-Tris gels (NUPAGE; Life technologies). Gels were fixed, stained with Coomassie Brilliant Blue G250 (Serva) as described in [78], and each lane was cut into eight fractions. The gel pieces were destained and proteins digested according to [62].

Liquid chromatography/mass spectrometry (LC/MS) was performed on a Q Exactive Plus (Thermo Scientific) coupled to an ultra-high performance Dionex Ultimate 3000 liquid chromatography unit (Thermo Scientific) via a Nanospray Flex Ion-Source (Thermo Scientific, Dreieich, Germany) essentially as described [62] with the following exceptions: peptides were separated on an in-house packed 2.4 µm Reprosil C18 resin (Dr. Maisch GmbH, Ammerbuch-Entringen Germany) picotip emitter tip (diameter 100 µm, 15 cm long, New Objectives) using a gradient from mobile phase A (4 % acetonitrile, 0.1 % formic acid) to 44 % mobile phase B (80 % acetonitrile, 0.1 % formic acid) for 30 min with a flow rate of 500 nl/min. The Full MS scan range was 300–2000 *m*/*z* with resolution of 70,000 at *m*/*z* 200. MS data were analyzed by MaxQuant 1.5.2.8 [19], which includes the MaxLFQ algorithm [18] for reliable, label-free protein quantitation. The enzyme specificity was set to trypsin, missed cleavages were limited to 2, variable modifications were N-terminal acetylation, oxidation of methionine, deamidation on asparagine and glutamine, and fixed modification was carbamidomethylation of cysteines. The mouse reference proteome database (download from Uniprot, June, 26th, 2015, 28,560 entries) was used to identify peptides and proteins. False discovery rate (FDR) for protein and peptides was 5 %.

### Quantitative mass spectrometric analyses of microdissected type I muscle fibers

Type I fibers in murine muscle were detected via immunofluorescence staining. Briefly, 10 μm cryosections were incubated with a primary antibody directed against myosin heavy chain I (NCL-MHCs Leica Microsystems, Wetzlar, Germany) in 1:40 dilution in PBS for 1 h at room temperature (RT) followed by an incubation with a secondary antibody conjugated with Texas Red (goat anti-mouse IgG antibody, Dianova, Hamburg, Germany, 1:400 in PBS) for 45 min at RT. Isolation of type I muscle fibers of the lower extremities was performed via laser microdissection. A total area of 250,000 μm^2^ of type I fibers was collected for each sample into tubes with 40 µl formic acid via laser microdissection (LMD6500, Leica Microsystems, Wetzlar, Germany). Samples were incubated for 30 min at RT, sonicated (35 kHz) for 5 min (RK31, BANDELIN electronic, Berlin, Germany) and centrifuged for 10 min (12,000×*g*, 4 °C). Prior tryptic digestion, the formic acid was removed by vacuum vaporization (rotational-vacuum-concentrator RVC2-25CD plus, Martin Christ GmbH, Osterode am Harz, Germany) and the collected tissue material was diluted in 50 mM ammonium bicarbonate (pH 7.8) to a final volume of 74.25 μl. Samples were reduced for 20 min at 56 °C by addition of DTT (final concentration of 6.7 mM) followed by alkylation with iodoacetamide (final concentration of 20 mM) for 15 min in the dark at RT. The pH was adjusted to 7.4 before adding 1 μl of 1 % Trypsin Enhancer ProteaseMAX Surfactant (Promega, Mannheim, Germany) in 50 mM ammonium bicarbonate (pH 7.8). Digestion was done by addition of trypsin (Serva Electrophoresis GmbH, Heidelberg, Germany) solution (1 μg/μl in 50 mM acetic acid) and overnight incubation at 37 °C. Digestion was stopped by addition of 4 μl of 2 % TFA, the samples were centrifuged for 30 min (16,000×*g*, 4 °C), and 63 µl of the supernatant were transferred into a new tube. 15 µl were used for each mass spectrometric analysis.

Label-free quantitative mass spectrometric analyses of tryptically digested microdissected type I muscle fibers was performed on an UltiMate 3000 RSLC nano LC system (Thermo Scientific, Bremen, Germany) as recently described [47]. The HPLC system was online-coupled to the nano ESI source of a Q Exactive mass spectrometer (Thermo Scientific, Bremen, Germany). The ESI-MS/MS analysis was performed as described [11].

Raw files were analyzed using Proteome Discoverer (ver. 1.4.1.14) (Thermo Fisher Scientific, Rockford, IL, USA). Peptide identification was performed by Mascot (version 2.5, Matrixscience, London UK) using the mus musculus complete proteome downloaded from UniProt (release 2015_5, containing 44,467 entries + decoys). Mass tolerance was set to 5 ppm for precursor ions and 20 mmu for fragment ions. As variable chemical modifications oxidation of methionine, phosphorylation of serine, threonine and tyrosine was used, carbamidomethylation of cysteine was set as fixed modification. One tryptic mis-cleavage was considered in the analysis. Confidence of peptide identification was determined using a protein inference algorithm (PIA) [74]. The filter cut-off for the identified peptides was set to targeted false discovery rates of <1 %.

Ion intensity-based label-free quantification was performed using Progenesis LC-MS software (Nonlinear Dynamics Ltd., Newcastle upon Tyne, UK) for data analysis. Raw files were imported and aligned. Quantified features were subsequently matched to peptide and protein identification by importing the search results from all samples generated using Mascot and PIA. Proteins quantified with an ANOVA *p* value <0.05 were considered to be significantly differentially expressed in one of the sample types.

### Quantitation of mitochondrial complexes and supercomplexes

Liquid nitrogen snap-frozen soleus muscles were homogenized in 200 µl homogenization buffer (50 mM sucrose, 20 mM sodium phosphate buffer pH 7.5, 1 mM EDTA, 2 mM 6-aminohexanoic acid) using an Ultra-Turrax (IKA, Staufen, Germany). The resulting suspension was diluted to a concentration of 10 mg tissue per 1 ml buffer and homogenized by 40 strokes of a motor-driven tightly fitting glass/Teflon Potter-Elvehjem homogenizer. Homogenates were centrifuged for 10 min at 10,000×*g*. Pellets corresponding to 10 mg muscle tissue containing nuclei as well as mitochondrial and other membranes were resuspended in 70 µl of buffer A (50 mM NaCl, 50 mM imidazole, pH 7, 1 mM EDTA, 2 mM 6-aminohexanoic acid), solubilized by addition of 8 µl 20 % digitonin (w/v in water), centrifuged for 10 min at 22,000×*g*, and the protein content of the supernatants were determined by Lowry’s assay [45]. Equal amounts of proteins per lane (10 µg for immunoblots, 30 µg for Coomassie Brilliant Blue and enzyme activity stained native gels) were loaded onto 3–16 % gradient gels following blue native electrophoresis (BNE) according to [78]. For quantitation of mitochondrial complexes, BN gels were either stained with Coomassie Brilliant Blue, blotted onto PVDF membranes for immunodetection, or used for an in gel complex I activity stain described by [81] with modifications according to [80].

### mtDNA long-range PCR

Total DNA was extracted from skeletal muscle specimens by column purification as described in the manual of QIAamp DNA Mini Kit (QIAGEN N.V., Venlo, Netherlands). Each sample was eluted twice in 200 µl elution buffer provided with the kit and was stored without freezing at 4 °C. To detect large-scale mtDNA deletions, long-range PCR analysis was performed in human and murine samples. Primer pair musMT2482F24 (5′-GTTCAACGATTAAAGTCCTACGTG-3′) and musMT1005R24 (5′-CCAGTATGCTTACCTTGTTACGAC-3′) was used for the murine and primer pair MT3137F26 (5′-GAGAAATAAGGCCTACTTCACAAAGC-3′) and MT45R22 (5′-TGGAGAGCTCCCGTGAGTGGTT-3′) for the human samples (first number, 5′ nucleotide of primer; F, forward primer; R, reverse primer; second number, length of primer). PCRs were performed with the LA Taq Hot Start DNA polymerase (Takara Bio Inc., Otsu, Japan) under the following conditions for the murine/human samples: 95 °C for 2.5 min, 10 cycles of 92 °C for 20 s and 66.8/68 °C for 5:30 min, 20 cycles of 92 °C for 25 s and 66.8/68 °C for 5:30 min, and 72 °C for 10 min.

### mtDNA deletion quantitation

Using total DNA prepared as described above, two different single molecule PCRs (smPCR) were performed for quantitative evaluation of the mtDNA deletions in murine samples. The first primer pair musMT2482F24 and musMT1005R24 amplified almost the entire mtDNA and the second primer pair musMT15795F21 (5′-TTCTTACTTCAGGGCCATCAA-3′) and musMT1005R24 a shorter region within the minor arc. The latter region of the mtDNA usually does not contain deletions and was used to determine the total amount of mtDNA molecules. For each genotype, the amount of large-scale mtDNA deletions was calculated according to [83] by dividing the quotient of the number of positive bands to its single molecule dilution in the deletion PCR with the quotient of the number of positive bands to its single molecule dilution in the total mtDNA amount PCR. Both PCRs were performed with LA Taq Hot Start DNA polymerase (Takara Bio Inc., Otsu, Japan) under the following conditions for the long/short mtDNA fragments: 95 °C for 2.5 min, 10 cycles of 92 °C for 20 s and 66.8/55 °C for 5:30/3 min, 20 cycles of 92 °C for 25 s and 66.8/55 °C for 5:30/3 min, and 72 °C for 10 min.

### mtDNA copy number

The mtDNA copy number was evaluated by quantitative real-time PCR (qPCR) using total DNA prepared as described above. For the detection of qPCR products, 2× SYBR Green qPCR Master Mix (Biotool, Munich, Germany) was used. The qPCR was performed with two different concentrations of the DNA sample, 10 and 20 ng/μl, in triplicates for each dilution. Primers amplifying the human mitochondrial fragment were MT3922F25 (5′-GAACTAGTCTCAGGCTTCAACATCG-3′) and MT4036R26 (5′-CTAGGAAGATTGTAGTGGTGAGGGTG-3′); primers for KCNJ10 (KIR4.1, potassium voltage-gated channel subfamily J member 10) were KIR835F19 (5′-GCGCAAAAGCCTCCTCATT-3′) and KIR903R19 (5′-CCTTCCTTGGTTTGGTGGG-3′). Primers amplifying the murine mitochondrial fragment were musMT553F23 (5′-GCCAGAGAACTACTAGCCATAGC-3′) and musMT668R23 (5′-AGCAAGAGATGGTGAGGTAGAGC-3′); primers for Kcnj13 (inward rectifier potassium channel 13) were mus4987F25 (5′-GGATGAGAGAGAGAAGCACAAGTGG-3′) and mus5140R25 (5′-CTGTATGACCAACCTTGGACATGAT-3′). The primer pairs for KCNJ10 and Kcnj13 were used to amplify single nuclear reference genes. All primer pairs were optimized and checked by PAGE. The qPCRs were performed under the following conditions for the human/murine samples: 95 °C for 7:00 min, 45 cycles of 95 °C for 15 s and 62.5/62.6 °C (nuc gene) or 62.5/64.6 °C (mito gene) for 1 min, 95 °C for 1 min and 55 °C for 1 min.

The obtained qPCR fluorescence data were analyzed by SigmaPlot (2001 for Windows version 7.0, Systat Software GmbH) and fitted with the Chapman sigmoidal regression curve [82]. Four parameters, i.e., *y*_0_, *a*, *b* and *c*, are determining the shape of the sigmoidal regression curve and the degree of exponential function. These parameters were provided by the software from the equation *y* = *y*_0_ + *a*(1 − *e*^−*bx*^)^*c*^. The *C*_t_ value is calculated at the inflection point of the sigmoidal curve from the equation *C*_t_ = ln(*c*)/*b*.

The *C*_t_ values for the single nuclear reference genes KCNJ10 and Kcnj13 were used for calculation of the mtDNA copy number according to [83]. First, the cycle number difference (Δ*C*_t_) was calculated by subtracting the *C*_t_ values of the mtDNA fragment (*C*_t_mito) of interest from the *C*_t_ values of the reference gene (*C*_t_nuc), Δ*C*_t_ = *C*_t_nuc − *C*_t_mito. Second, the copy number (CN) of the mtDNA relative to the diploid single nuclear gene was calculated as CN = 2 × 2^Δ*C*t^.

To validate the qPCR results, the PCR amplification efficiency of each primer pair was determined as recommended (PCR efficiency = (10^−1/slope^ − 1) × 100; [6]), which were 97 % for MT3922F25/MT4036R26, 104 % for KIR835F19/KIR903R19 (KCNJ10), 103 % for musMT553F23/musMT668R23, and 95 % for mus4987F25/mus5140R25 (Kcnj13). The calibration curves for each primer pair amplifying DNA from human or murine control samples (serial dilution concentrations 3.2, 1.6, 0.16, 0.016 ng/µl) proved the excellent linearity of the qPCRs (data not shown). The NTC *C*_t_ values for each primer pair were as follows: 145/no curve/no curve (negative values) for MT3922F25/MT4036R26, 40.49/40.49/40.98 for KIR835F19/KIR903R19 (KCNJ10), 40.8/97.7/50.4 for musMT553F23/musMT668R23, and 40.09/43.95/38.99 for mus4987F25/mus5140R25 (Kcnj13).

### Data analysis

Data analyses and statistical evaluations were performed using Excel 2010 (Microsoft); the Kruskal–Wallis one-way analysis of variance and the Mann–Whitney *U* (Wilcoxon rank-sum) statistical tests were done using the Excel add-in “Real Statistics Resource Pack” version 3.1.2 by Charles Zaiontz available at http://www.real-statistics.com/. The number of independent experiments, number of technical replicates, mean values, standard errors, type of statistical test, and significance levels are indicated in the “Results” section or Figure legends. Final assembly and preparation of all figures for publication was done using Corel Draw Graphics Suite X7.

## Results

### Focal depletion of mitochondria in human and murine desminopathies

**Fig. 1:**
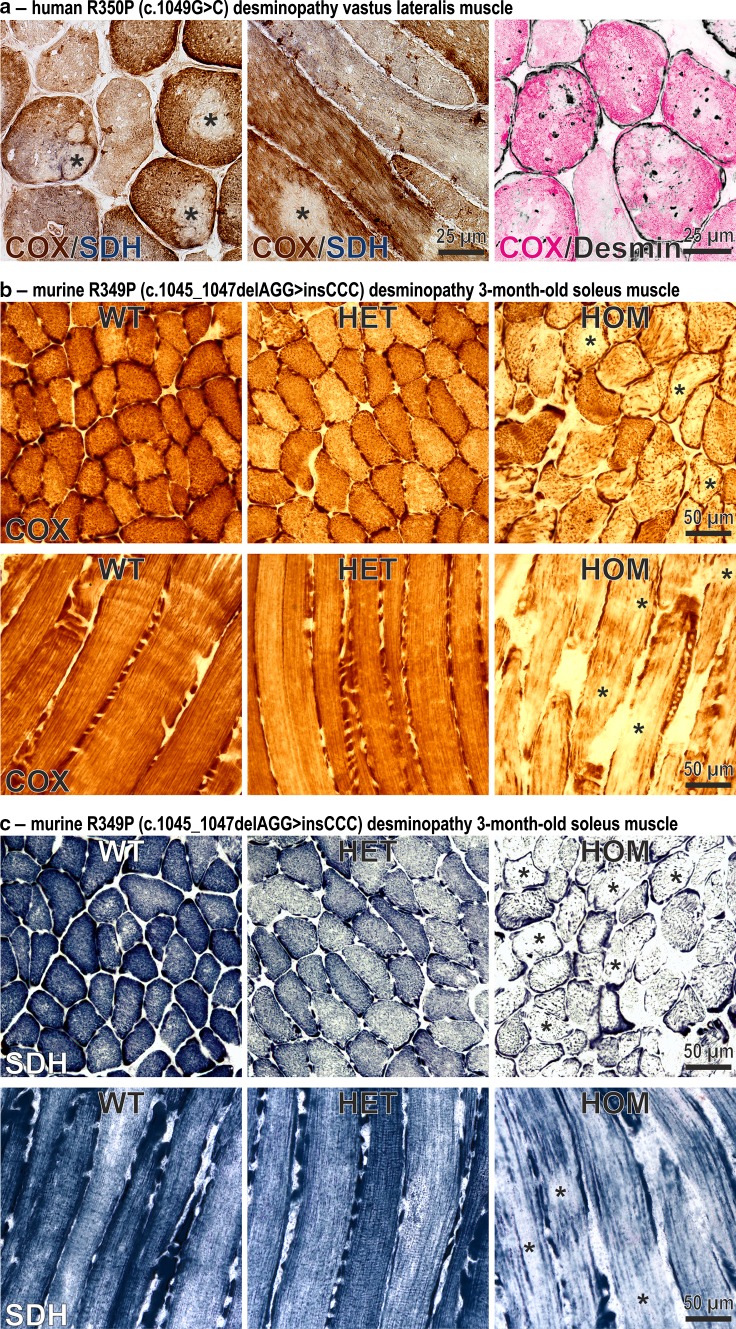
Focal depletion of mitochondria in skeletal muscles of a human R350P desminopathy patient and R349P desmin knock-in mice. **a** Cytochrome *C* oxidase (COX, *brown*) and succinate dehydrogenase (SDH, *blue*) double-stains of transverse and longitudinal cryosections from a German patient harboring the heterozygous R350P (c.1049G>C) desmin missense mutation [3]. Note the multiple rubbed-out areas (*asterisks*) devoid of COX and SDH enzyme activities demonstrating the absence of mitochondria. The *right image* represents a false color representation of two superimposed serial cryosections stained for COX (*magenta*) and desmin (*black*). Sarcoplasmic desmin-positive protein aggregates clearly display a distribution which is independent from the mitochondrial lesion pathology. **b**, **c** COX (**b**) and SDH (**c**) stains of transverse and longitudinal cryosections from 3-month-old R349P (c.1045_1047delAGG>insCCC) desmin knock-in mice. Fibers of homozygote (HOM) animals display large and small areas of diminished enzyme stains (*asterisks*). Furthermore, muscle fibers in homozygote animals show an abnormal, thread-like distribution of mitochondria. Heterozygous (HET) mice showed no overt pathology as compared to wild-type (WT) littermates

Human skeletal muscle specimens from a patient with a heterozygous R350P (c.1049G>C) desmin mutation [3] were analyzed by Gomori trichrome (not shown), COX, SDH, and desmin stains, respectively. Though no ragged-red fibers or completely COX-negative/SDH-positive fibers were detected, multiple fibers displayed rubbed-out areas with attenuated or even absent COX and SDH enzymatic activities (Fig. 1a, left and middle panels, asterisks) indicating focal depletion of mitochondria in multiple muscle fibers. To address the question whether the presence of desmin-positive protein aggregates correlates to sarcoplasmic areas with mitochondria depletion, we performed COX enzyme activity and desmin immunofluorescence stains on different serial transverse cryosections. Superimposed images indicated that the distribution of desmin protein aggregates is independent from the presence of mitochondrial rubbed-out lesions (Fig. 1a, right panel). Notably, this mitochondrial depletion pattern in the human desminopathy was also detected in skeletal muscle of our homozygous R349P (c.1045_1047delAGG>insCCC) desmin knock-in mice (Fig. 1b, c, asterisks). In contrast, no obvious mitochondrial alterations were observed in heterozygous animals. The sections of murine soleus muscle used for these stains were derived from 3-month-old hetero- and homozygous mice, which at this stage did not exhibit any signs of muscle weakness [16].

**Fig. 2:**
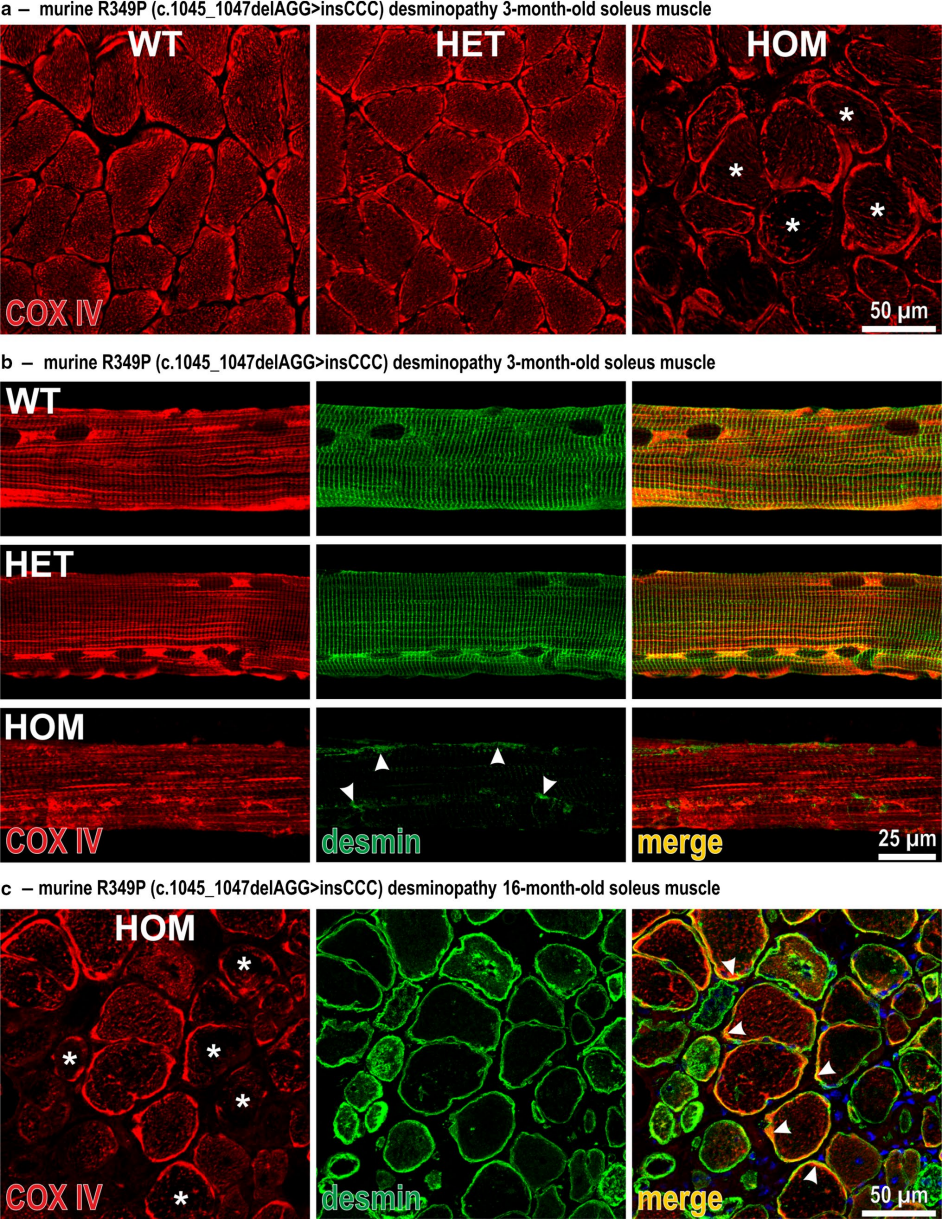
Aberrant subcellular distribution of mitochondria and desmin intermediate filament pathology in R349P desmin knock-in mice. **a** Indirect immunofluorescence analysis of COX IV in transverse sections of 3-month-old soleus muscle from wild-type (WT), heterozygous (HET) and homozygous (HOM) animals. Note the markedly reduced sarcoplasmic COX IV signal in multiple homozygous fibers (*asterisks*). **b** COX IV and desmin double-immunostains of isolated soleus muscle fibers. In contrast to a regular cross-striated staining pattern of both COX IV and desmin in wild-type and heterozygous R349P desmin knock-in mice, fibers from homozygous animals displayed a severe derangement of the mitochondria distribution, markedly reduced overall desmin signal intensities, and the presence of small subsarcolemmal and sarcoplasmic desmin protein aggregates (*arrowheads*). **c** COX IV and desmin double-immunostains of transverse sections of soleus muscle from 16-month-old homozygous mice. Note the presence of multiple fibers with virtual absence of the sarcoplasmic COX IV signal (*asterisks*) and the partial co-localization of mitochondria and mutant desmin in the subsarcolemmal region (*arrowheads*)

In a next step, we analyzed the subcellular distribution of mitochondria in cross-sections of murine soleus muscle of 3-month-old R349P desmin knock-in animals by indirect immunofluorescence imaging. Compared to wild-type and heterozygous mice, COX IV immunostaining patterns in homozygous mice exhibited markedly reduced fluorescence signal intensities in the intermyofibrillar space in the vast majority of fibers (Fig. 2a, asterisks), whereas the subsarcolemmal COX IV signal intensity remained unchanged. Furthermore, single fiber preparations from soleus muscles were used to study the relationship between the subcellular distribution of mitochondria and the desmin intermediate filament network. While fibers from heterozygous mice did not show any overt abnormalities, fibers from homozygous mice displayed a marked disorder of the COX IV signal pattern with loss of its normal cross-striation arrangement (Fig. 2b). This altered organization was accompanied by a substantial change in the desmin staining pattern comprising a marked reduction of the overall signal intensity, an extensive loss of its cross-striation pattern, and the presence of subsarcolemmal and sarcoplasmic desmin-positive protein aggregates (Fig. 2b, arrowheads). In aged soleus muscle of 16-month-old animals, the aberrant COX IV distribution was even more pronounced with multiple fibers displaying a virtually absent sarcoplasmic COX IV signal (Fig. 2c, asterisks). Herein, the R349P mutant desmin showed a partial co-localization with mitochondria in the subsarcolemmal region (Fig. 2c, arrowheads).

### Mitochondrial pathology at the ultrastructural level

**Fig. 3:**
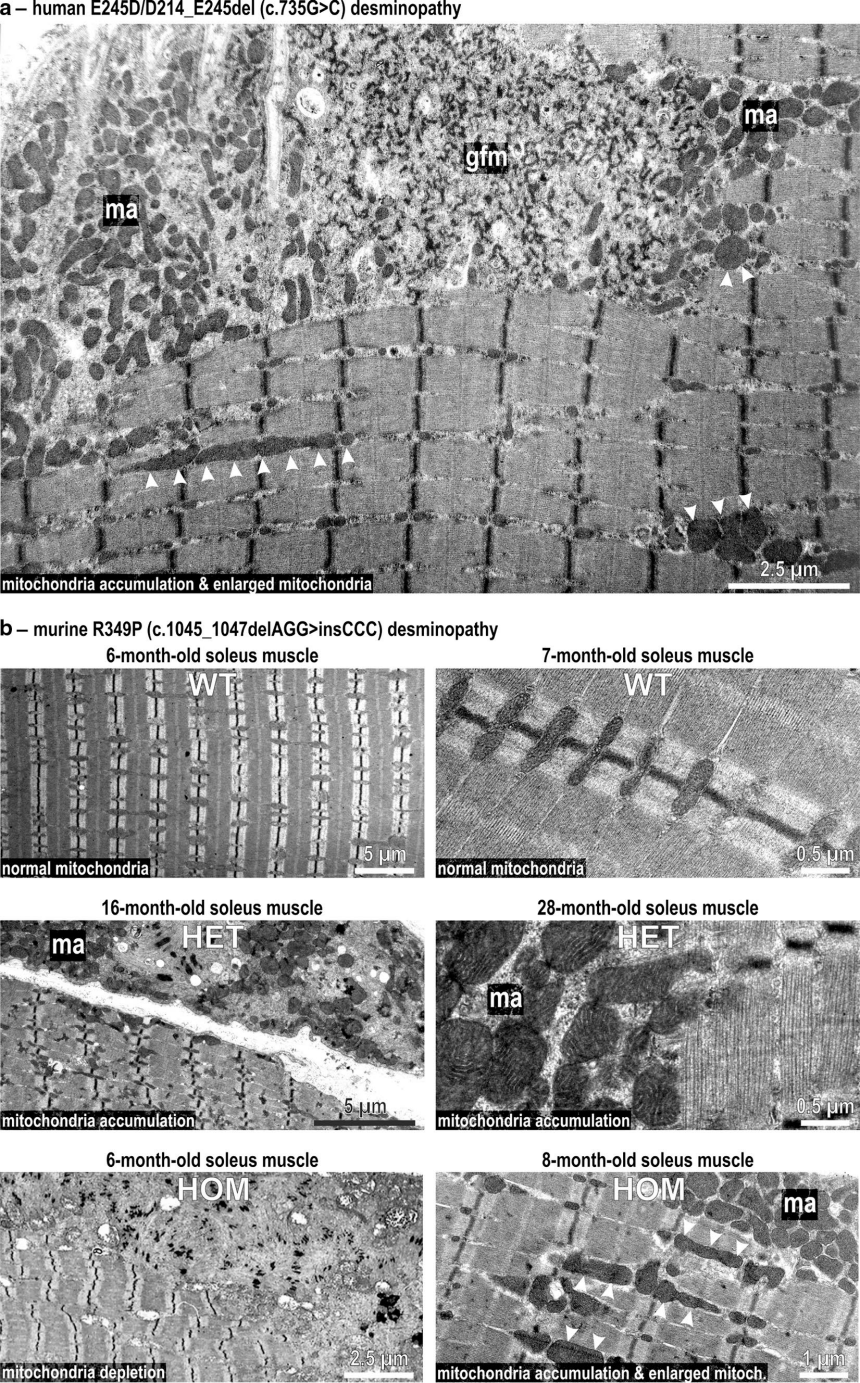
Electron microscopic visualization of mitochondrial pathology in human and murine desminopathies. **a** Granulofilamentous material (gfm), subsarcolemmal accumulation of mitochondria (ma), and multiple enlarged mitochondria (*arrowheads*) in skeletal muscle tissue from a German patient harboring a heterozygous E245D/D214_E245del (c.735G>C) desmin mutation [14]. **b** *Upper panel* normal mitochondria regularly positioned adjacent to myofibrillar Z-discs in murine wild-type (WT) soleus muscle. *Middle panel* aged heterozygous (HET) R349P desmin knock-in mice occasionally displayed focal accumulation (ma) of normal and enlarged mitochondria. *Lower panel* homozygous (HOM) mice, the *left image* illustrates the depletion of mitochondria in a large sarcoplasmic area comprising normal myofibrils and myofibrillar remnants. The *right image* depicts focal mitochondrial accumulation (ma) and enlarged mitochondria (*arrowheads*) in the intermyofibrillar space

In our electron microscopic analysis of skeletal muscle tissue from desminopathy patients, we discovered that in the case of the heterozygous c.735G>C desmin mutation that leads to the expression of two mutant desmin proteins, E245D and D214_E245del [14], areas with a subsarcolemmal accumulation of mitochondria (Fig. 3a, ma) did prominently localize in close association with pathological protein aggregates composed of granulofilamentous material (Fig. 3a, gfm). Moreover, enlarged mitochondria in the subsarcolemmal and intermyofibrillar regions were present (Fig. 3a, arrowheads). To assess whether these mitochondrial abnormalities observed in human desminopathy muscle are also present in our R349P desmin knock-in mice, corresponding electron microscopy analyses of aged heterozygous R349P desmin knock-in mice were performed, occasionally revealing abnormal fibers with remnants of degenerating myofibrils that were interspaced with an accumulation of enlarged mitochondria (Fig. 3b, middle panels). In keeping with our light microscopy data, the mitochondrial changes-comprising areas with depletion or accumulation of mitochondria were present in the vast majority of muscle fibers derived from 6 to 8-month-old homozygous R349P desmin knock-in mice (Fig. 3b, bottom panels). In addition, enlarged mitochondria were frequently observed (Fig. 3b, bottom panels, arrowheads).

### Expression of R349P desmin impacts the mitochondrial proteome and the respiratory chain complex organization

**Fig. 4:**
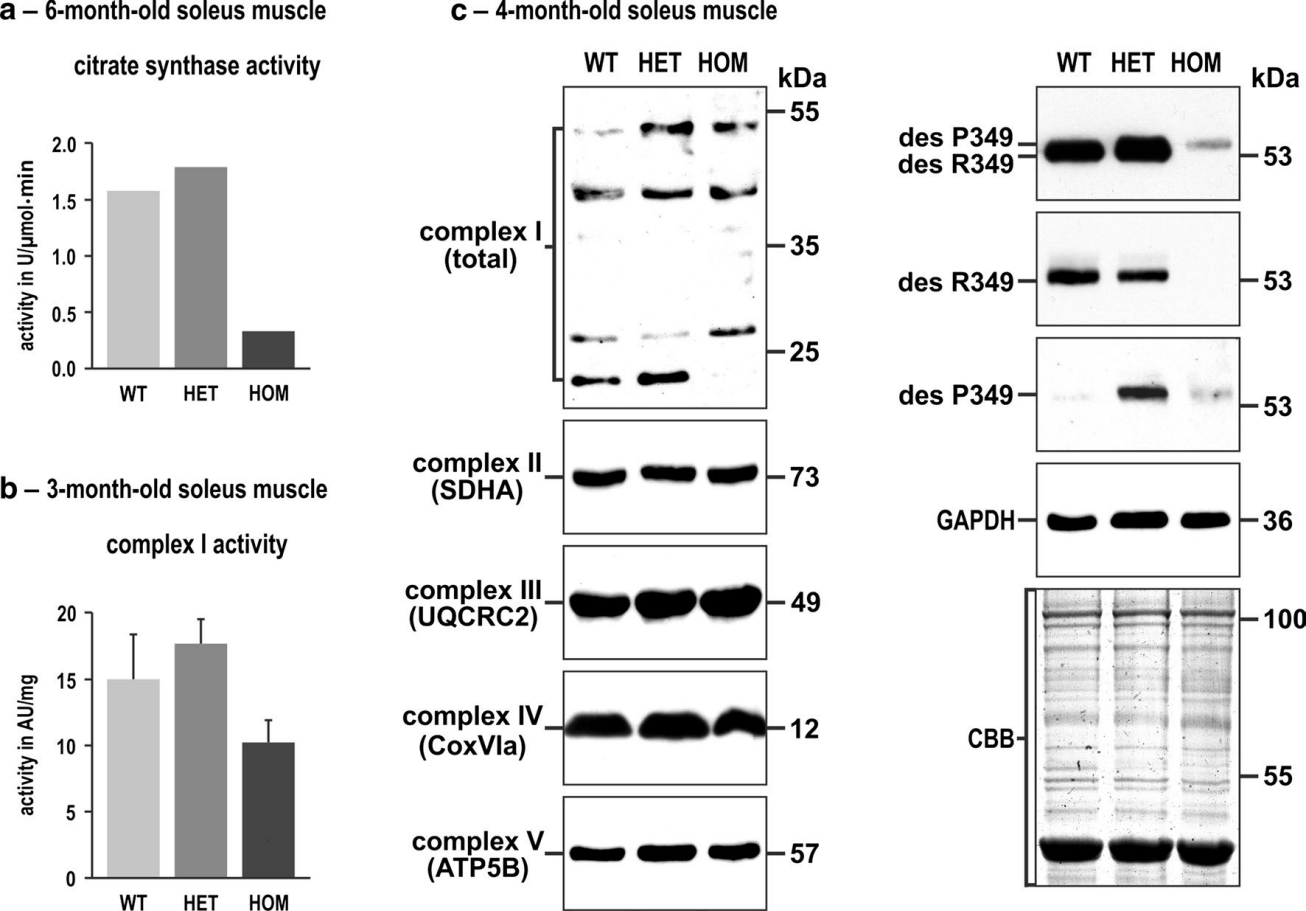
Citrate synthase and complex I activities and expression levels of single subunit proteins of respiratory complexes I–V in R349P desmin knock-in mice. **a** Determination of the citrate synthase activity by a spectrophotometric assay using identical amounts of total protein extracts. Note the marked activity reduction in homozygous mice. For this approach, soleus muscles obtained from five mice of each genotype (WT, HET, HOM) were pooled and subjected to four-time repeated measurements.* Column chart* shows mean values of these technical replicates. **b** Determination of complex I activity by a colorimetric assay normalized to muscle weight. Though this analysis, performed in duplicate on non-pooled samples derived from three animals per genotype, showed a reduction of complex I activity in homozygous mice, the data failed to reach statistical significance. An additional normalization of the obtained complex I values to the citrate synthase values resulted in similar activity levels for the three genotypes (data not shown). *Column chart* shows mean values and standard errors. **c** Immunoblotting using antibodies directed against specific complex I–V proteins revealed increased, decreased, and missing signals of complex I subunit proteins in heterozygous and homozygous mice, whereas the signal intensities of complex II–V subunit proteins showed no obvious differences. Desmin immunoblotting confirmed a markedly decreased level of mutant desmin in homozygous mice. In heterozygous mice the obtained signal is composed of the signals from both R349 wild-type and P349 mutant desmin, as previously demonstrated by high resolution SDS-PAGE and immunoblotting [16]. GAPDH immunoblotting and Coomassie stained SDS-PAGE gels were used as loading controls

In a next step, we assessed mitochondrial dysfunction on the biochemical level. Since no sufficient amounts of human desminopathy biopsy material were available, these experiments were limited to murine muscle tissue. Measurements of citrate synthase activity—a standard marker for intact mitochondria content—revealed a reduction by 79 % in soleus muscle tissue homogenates derived from homozygous R349P desmin knock-in mice as compared to wild-type lysates (Fig. 4a). Subsequently, we analyzed enzyme activities of respiratory chain complexes I (Fig. 4b) and IV (data not shown) in tissue homogenates, both normalized to muscle weight and to the citrate synthase activity. However, these experiments showed no statistically significant differences between the three genotypes analyzed.

To quantitate the presence of both mtDNA and nuclear DNA-encoded protein subunits of the mitochondrial complexes I–V, we employed immunoblotting as well as a proteomic approach. For immunoblotting, identical amounts of total protein extracts from soleus muscle tissue were analyzed using antibodies directed against individual, nuclear DNA-encoded subunits of complexes I–V (Fig. 4c). This analysis revealed marked alterations in complex I that was detected by an anti-serum recognizing multiple subunits of complex I [8], while no overt changes for the complexes II–V were noted (Fig. 4c).

To address complex I–V subunit composition in more detail including mtDNA-encoded proteins, we performed label-free quantitative mass spectrometry analyses based on identical amounts of total protein extracts derived from either whole soleus muscles or type I fibers from lower extremity muscles (Fig. S1). The soleus muscle, which is rich in type I fibers, was chosen because it was found to be more susceptible to damage by mutant desmin than muscle groups containing predominantly type II fibers [16]. Though the soleus muscle is composed of a relatively high amount of mitochondria-rich type I fibers (approx. 50 %), it still contains a significant number of type II fibers. Thus, to obtain samples of pure type I fibers, they were collected by laser microdissection from lower extremity muscles. Our analyses revealed specific differences related to the method of sample preparation, the knock-in mouse genotype, the individual mitochondrial respiratory chain complex, and the mitochondrial or nuclear origin of the individual protein subunit. Since the yield of identified respiratory chain proteins was much higher in soleus muscle homogenates than in the collected type I fibers, the former data gives a more comprehensive insight into the aberrant mitochondrial proteome. The analysis of mtDNA-encoded proteins in heterozygous mice showed a marked reduction (mean fold change as compared to wild-type: 0.64) of their abundance, whereas the levels of these proteins in homozygous mice mostly were unchanged (mean fold change: 0.97). In contrast, the vast majority of nuclear-encoded proteins from all five respiratory complexes was found to be markedly down-regulated in both hetero- and homozygous genotypes (mean fold changes: het, complex I, 0.68; hom, complex I, 0.65; het, complex II, 0.52; hom, complex II, 0.75; het, complex III, 0.69; hom, complex III, 0.63; het, complex IV, 0.62; hom, complex IV, 0.79; het, complex V, 0.66; hom, complex V, 0.70). Thus, the quantitative proteomic analysis clearly demonstrated a mean overall decrease of the mtDNA- and nuclear DNA-encoded respiratory chain proteins of 34 and 30 % in heterozygous and homozygous soleus muscles, respectively. In agreement with our immunoblotting results (Fig. 4c, right panel; and [16]), the quantitative mass spectrometry data showed that the R349P mutant desmin protein levels were markedly reduced to 10 % in homozygous mice.

**Fig. 5:**
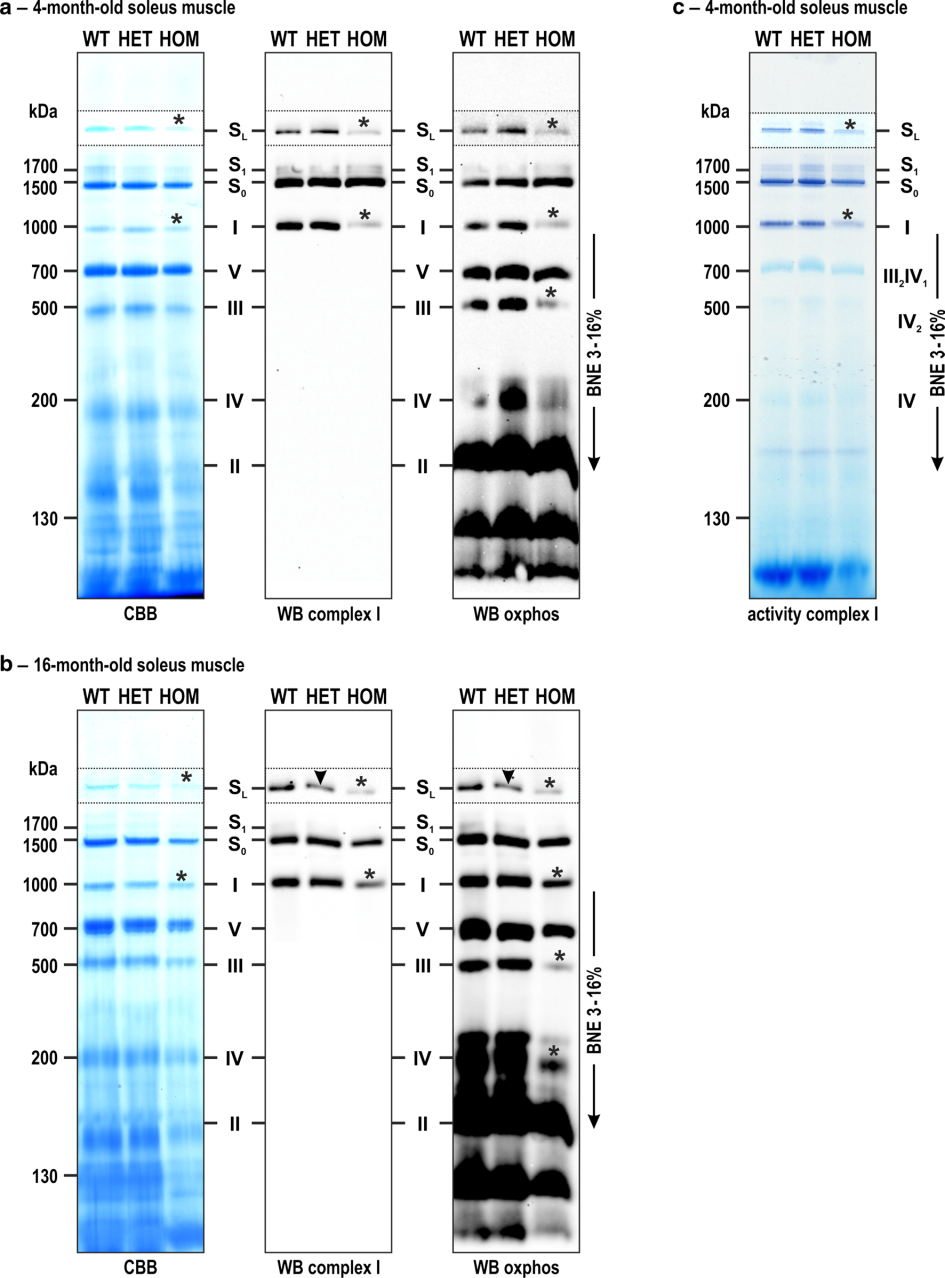
Alterations of the respiratory chain and oxidative phosphorylation complex assembly as assessed by blue native gel electrophoresis. Protein complexes derived from identical amounts of enriched mitochondria from 4- and 16-month-old wild-type (WT), heterozygous (HET), and homozygous (HOM) R349P desmin knock-in littermates were separated by blue native electrophoresis in 3–16 % acrylamide gradient gels. **a**, **b** Gels were Coomassie stained or used for immunoblotting against complex I subunits or “OXPHOS multiplex” using an antibody mix against all respiratory chain and oxidative phosphorylation complexes. Note the reduced amounts of complex I as free component and as part of the large supercomplex *S*_L_ in young and aged homozygous mice (*asterisks*), and as part of supercomplex *S*_L_ in aged heterozygous mice (*arrowheads*). **c** A native gel as in *panel* (**a**) was used for complex I activity visualization, which demonstrated a corresponding reduction of the enzymatic activity. Mitochondrial complexes are indicated: I, complex I; II, complex II; III, dimeric complex III; IV, complex IV; V, complex V; *S*_0-1_, respiratory supercomplexes containing complex I, dimeric complex III and 0 to 1 copy of complex IV; *S*_L_, larger supercomplex containing two complex I and one complex III dimer

Beyond changes in the levels of single respiratory chain and oxidative phosphorylation complex proteins, we addressed putative alterations with respect to the sizes and amounts of mitochondrial protein complexes I–V by native gel electrophoresis in conjunction with Coomassie Brilliant Blue staining, immunoblotting, and specific complex activity stains. For this purpose, mitochondria were enriched from soleus muscle tissue specimens derived from 4-month as well as 16-month-old animals, and identical amounts of mitochondria were subjected to protein separation by blue native electrophoresis (BNE). Coomassie Brilliant Blue stained BN gels already indicated a slight decrease in the amount of complex I both as free component and as part of the large supercomplex *S*_L_ in homozygous soleus muscles of 4-month-old animals (Fig. 5a, left panel, asterisks). This impression was substantiated by immunoblotting of complex I, which depicted a far more pronounced decrease (Fig. 5a, middle panel, asterisks). To determine the composition and stoichiometry of the respiratory supercomplexes, bands from BN gels containing the complexes I, III_2_, IV, *S*_0_, *S*_1_ and *S*_L_ were further analyzed by quantitative mass spectrometry. This analysis revealed that the respiratory supercomplex *S*_0_ (apparent native mass 1378 kDa) is formed by complex I and a dimer of complex III, *S*_1_ (apparent native mass 1590 kDa) by complex I, a dimer of complex III and complex IV, and *S*_L_ (apparent native mass 2284 kDa) by two complex I and a dimer of complex III. Multiplex immunoblotting with antibodies directed against subunits of all complexes additionally revealed decreased signal intensities for the individual complex III (Fig. 5a, right panel, asterisks). The respiratory supercomplexes *S*_0_ and *S*_1_ were present in comparable amounts in all three genotypes. When a similar analysis was performed using 16-month-old desmin knock-in mice, decreased amounts of complexes *S*_L_, I, III, and IV in homozygous compared to wild-type soleus muscles were detected by Coomassie Brilliant Blue stained BN-PAGE gels as well as the corresponding complex I and multiplex immunoblots (Fig. 5b, asterisks). Notably, in the aged heterozygous mice we additionally detected a decreased signal intensity of complex I as part of the supercomplex *S*_L_ (Fig. 5b, middle and right panels, arrowheads). Furthermore, our gel-based complexes activity stains demonstrated that the decreased amounts of formed complexes I and *S*_L_ (Fig. 5c, asterisks) are associated with a corresponding, lower enzyme activity.

### Expression of mutant desmin leads to large-scale mtDNA deletions and reduced mtDNA copy numbers

**Fig. 6:**
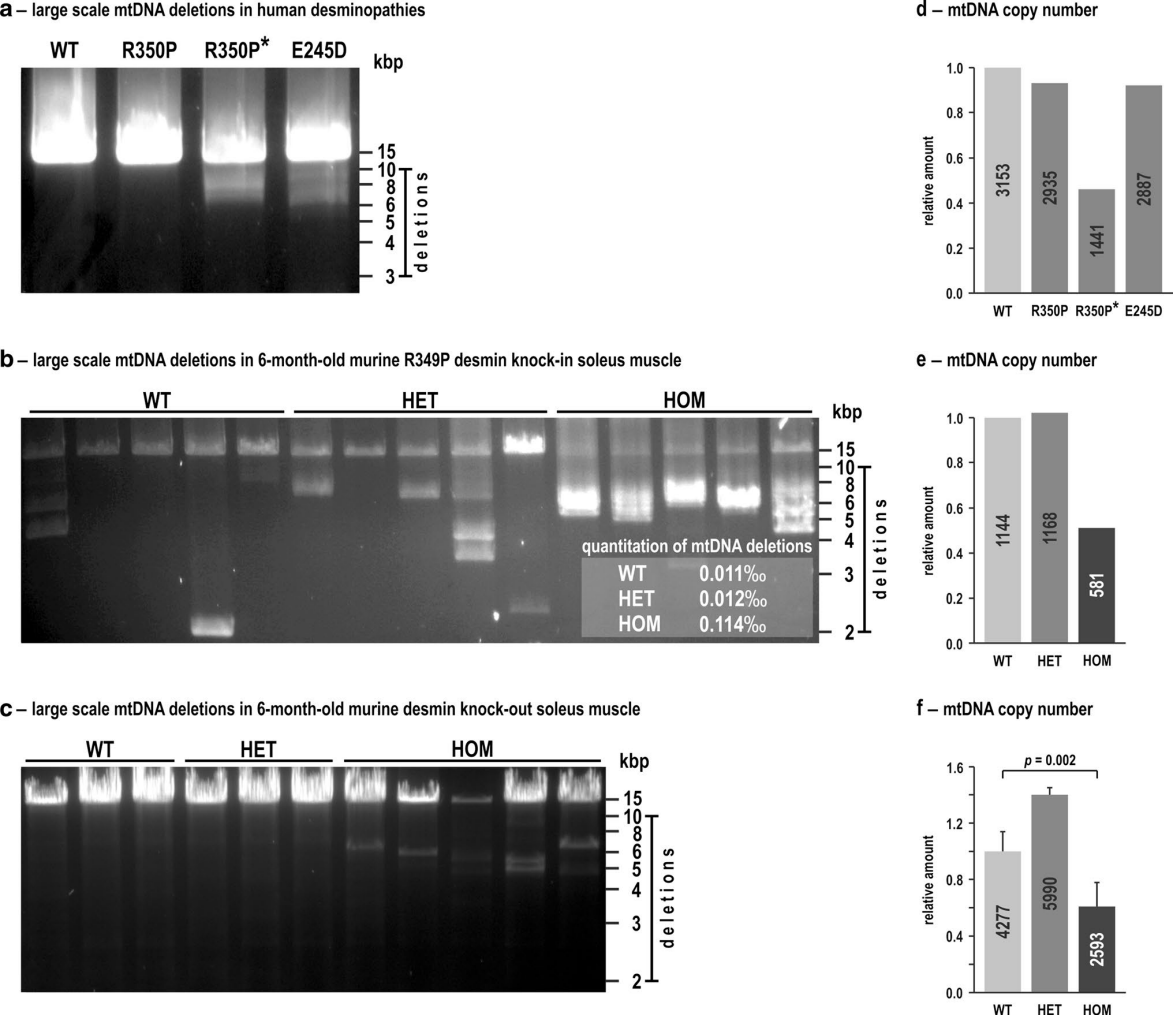
Large-scale mtDNA deletions and reduced mtDNA copy numbers in human and murine desminopathies. **a** Total DNA extracted from human skeletal muscle specimens derived from a normal control (WT) and three different desminopathies was used for long-range PCRs to detect large-scale mtDNA deletions. Note the presence of large-scale mtDNA deletions visible as additional bands between 10 and 6 kbp in cases R350P* and E245D/D214_E245del. **b** Total DNA extracted from soleus muscles (same pooled material derived from five mice per genotype as used in Figs. 4a and S1) was used for five-times repeated long-range PCRs to detect large-scale mtDNA deletions in 6-month-old wild-type (WT), heterozygous (HET) and homozygous (HOM) R349P desmin knock-in mouse littermates. *Inset*, amount of large-scale mtDNA deletions quantitated by a single molecule PCR (smPCR) approach. The* numbers* shown are mean values from three technical replicates using the pooled material from five mice per genotype. **c** Long-range PCRs from total DNA extracted from soleus muscles of 6-month-old wild-type (WT, three individual mice), heterozygous (HET, three individual mice) and homozygous (HOM, five individual mice) desmin knock-out mouse littermates. While no large-scale mtDNA deletions were detected in wild-type and heterozygous mice, they were present in homozygous animals. Note, however, that the degree of mtDNA deletions was markedly lower as compared to homozygous R349P desmin knock-in mice, in which the shortened mtDNA bands were the most prominent species. **d**–**f** The mtDNA copy numbers (absolute values indicated inside the *columns*) were determined by quantitative real-time PCR in the human desminopathy (**d**), murine R349P desmin knock-in (**e**), and murine desmin knock-out (**f**) skeletal muscle specimens as used in (**a**–**c**). *Column charts* indicate mean relative values from three technical replicates (**d**, **e**), and mean relative values and standard errors of the independent biological samples (**f**) with wild-type values set as 1. A statistically significant difference between the three genotypes in (**f**) was first analyzed using the Kruskal–Wallis one-way analysis of variance; post-hoc analyses were performed using the Mann–Whitney *U* test, and the single *p* value for the significant difference between WT and HOM is indicated

To address putative mtDNA damage, we performed long-range PCRs using DNA extracted from skeletal muscles derived from three human desminopathy patients as well as from 6-month-old wild-type, heterozygous and homozygous littermates of our R349P desmin knock-in mice. In all three human specimens, these PCR experiments resulted in the amplification of the full-length mtDNA (Fig. 6a). The samples of two out of the three desminopathy patients revealed the additional presence of large-scale mtDNA deletions. These deletions were observed in one previously reported patient harboring the heterozygous E245D/D214_E245del (c.735G>C) desmin mutation (Fig. 6a, fourth column), who underwent muscle biopsy at the age of 38 years [14], and a second patient with a R350P (c.1049G>C) desmin mutation. Here, it is noteworthy that the R350P patient with the large-scale mtDNA deletions (father; 52 years of age at biopsy; Fig. 6a, third column, with asterisk) and the one without large-scale mtDNA deletions (son; 28 years of age at biopsy; Fig. 6a, second column) are members of the same previously reported family [3].

PCR analyses of wild-type mice resulted in the amplification of the full-length mtDNA, however, additional mtDNA species with large-scale deletions of different sizes were detected (Fig. 6b). A similar pattern was observed in heterozygous mice, which also showed these additional, rearranged mtDNA species. In contrast, the analysis of homozygous animals showed a clearly different picture, with only traces of the amplified full-length mtDNA in conjunction with prominent signals corresponding to smaller mtDNA species (Fig. 6b). For quantitative evaluation of the mtDNA deletions, two different single molecule PCR (smPCR) series were performed using a first primer pair amplifying almost the entire mtDNA and a second primer pair amplifying a shorter region within the minor arc. The latter region of the mtDNA usually does not contain deletions and was used to determine the total amount of mtDNA molecules. For each genotype, the amount of large-scale mtDNA deletions was calculated according to [83] by dividing the quotient of the number of positive bands to its single molecule dilution in the deletion PCR with the quotient of the number of positive bands to its single molecule dilution in the total mtDNA amount PCR. This quantitation demonstrated a tenfold increased amount of large-scale mtDNA deletion species in homozygous mice as compared to both wild-type and heterozygous littermates (Fig. 6b, inset). In principle, the mtDNA pathology in homozygous R349P desmin knock-in mice may either be attributed to the presence of point-mutated desmin, the lack of wild-type desmin, or both. To address this issue, we additionally included age-matched, 6-month-old desmin knock-out mice [40] (for review see [15]) at this stage of analysis. Here, we found neither mtDNA deletions in wild-type nor in heterozygous mice, while homozygous desmin knock-out mice were found to display some mtDNA deletions (Fig. 6c). The degree of mtDNA deletions, however, was remarkably lower as compared to the homozygous R349P desmin knock-in mice, in which the shortened mtDNA bands were the most prominent mtDNA species (Fig. 6b). Lastly, we studied the mtDNA copy numbers in the same skeletal muscle tissue specimens derived from the human desminopathy patients as well as the R349P desmin knock-in and desmin knock-out mice. A marked reduction of the mtDNA copy number was observed in the aged R350P desminopathy patient with the multiple mtDNA deletions (Fig. 6d), and in homozygous desmin knock-in (Fig. 6e) and homozygous desmin knock-out mice (Fig. 6f).

## Discussion

Mitochondrial dysfunction has been described in a wide variety of human degenerative diseases affecting the central nervous system [4, 5, 17, 36, 43, 52, 61, 72] and striated muscle tissue [35, 57]. In the context of myofibrillar myopathies, mitochondrial pathology has been reported in patients with mutations in filamin-C [34, 37, 46], myotilin [34, 59], ZASP [34], FHL1 [48], plectin [69, 77], and desmin [3, 67, 68]. However, the functional relationship between the expression of a mutant, disease-causing protein and the associated mitochondrial dysfunction remains largely enigmatic in the majority of these disease entities.

### A strong link for desminopathy and mitochondrial pathology

In the context of desminopathies, a direct functional link between desmin pathology and mitochondrial dysfunction can be formulated based on the observations that mitochondria co-localize with the desmin intermediate filament network [63] and that desmin knock-out mice display defects in the morphology and positioning as well as in the respiratory enzyme function of mitochondria in striated muscle tissue [25, 44, 54]. However, the consequence of the expression of mutant desmin proteins on mitochondria is still an unresolved issue. To address this issue in a mechanistic perspective, we analyzed skeletal muscle tissue specimens derived from the R349P desmin knock-in mice and human desminopathy patients harboring heterozygous *DES* mutations.

In a first step, we compared SDH and COX stains of skeletal muscle from a human R350P (c.1049G>C) desminopathy patient with those from heterozygous and homozygous mice that carry the orthologous R349P (c.1045_1047delAGG>insCCC) desmin mutation. In the human biopsy, multiple fibers with large sarcoplasmic areas devoid of SDH and COX enzyme activities (“rubbed-out” lesions) were noted. However, COX-negative and ragged-red fibers, which were previously reported to occur in sporadic and familial desminopathies [32, 50], were not found in our analysis. Evidence for mitochondrial abnormalities comprising large and small areas of diminished COX and SDH enzyme stains were also present in the homozygous R349P desmin knock-in mice, which solely express the mutant desmin. In addition, our immunofluorescence analysis of soleus muscle tissue and isolated fibers showed a reduced sarcoplasmic COX IV signal intensity. The ultrastructural analysis of human E245D/D214_E245del (c.735G>C) desminopathy muscle provided additional signs of mitochondrial pathology with accumulated and enlarged mitochondria. In line with our histochemistry results, our ultrastructural analysis of homozygous mice depicted areas with depletion or accumulation of mitochondria as well as enlarged mitochondria. Mitochondria with paracrystalline inclusions, which have been demonstrated in a previous report on human desminopathies [75], could not be detected.

### Mitochondrial biochemistry in desminopathy

In a recent study, the analysis of citrate synthase-normalized respiratory chain enzyme activities in skeletal muscle homogenates of a heterozygous R350P desminopathy patient, who was not related to both R350P desmin patients included in this study, gave normal results [34]. In our previous analysis of a desminopathy patient harboring a heterozygous single codon deletion (K240del; c.720_722delGAA), the analysis of respiratory chain enzymes in skeletal muscle homogenates also showed normal absolute and citrate synthase-normalized activities of complexes I and IV, but the analysis of single saponin-permeabilized skeletal muscle fibers finally depicted an in vivo inhibition of the complex I activity [67, 68]. However, in a patient with a heterozygous S13F (c.38C>T) desmin mutation, more drastic mitochondrial enzyme activity changes including a significant reduction of the citrate synthase activity, an increase of complexes II to IV activities, and a loss of complex I activity were reported [50].

In line with the light and electron microscopy findings indicating a depletion of mitochondria, measurement of the citrate synthase activity revealed that it was strongly reduced in soleus muscle from homozygous R349P desmin knock-in mice. In contrast, neither complex I nor IV enzyme activities were significantly changed in hetero- and homozygous R349P desmin knock-in mice. Our mass spectrometry-based quantitation of nuclear and mtDNA-encoded complex I–V protein subunits showed a marked reduction of their abundance in R349P desmin knock-in mice. The analysis of the respiratory chain and oxidative phosphorylation complexes by native gel electrophoresis demonstrated reduced amounts of assembled complexes I, III, and IV in homozygous mice. We additionally addressed the now widely accepted notion that complexes I, III and IV form stoichiometric and stable units called supercomplexes or respirasomes [65]. The formation of supercomplexes is thought to be essential for the stability of the individual complexes [1, 10, 21], efficient substrate channeling [27], and for the prevention of reactive oxygen species generation [49]. Our study provides also evidence for the formation of higher molecular complexes (*S*_L_), which are formed by higher organization of the respiratory supercomplexes into superassemblies [58, 79]. Notably, already young homozygous R349P desmin knock-in mice exhibited a reduced amount of complex I in the *S*_L_ superassembly, whereas this deviation in the heterozygous state became only apparent in aged animals. This specific reduction of *S*_L_ indicates that only the higher assembly of mitochondrial respiratory supercomplexes [71, 79] is destabilized by the presence of mutant desmin.

### The state of mitochondrial DNA

Last but not least, we addressed the extent of the mtDNA damage in three human desminopathies and in our desminopathy mouse model. Long-range PCRs detected large-scale mtDNA deletions in one of the two R350P (c.1049G>C) desmin patients from the same family. Moreover, the E245D/D214_E245del (c.735G>C) desminopathy patient showed large-scale mtDNA deletions. In another, unrelated R350P desminopathy patient [34] and the aforementioned S13F (c.38C>T) desminopathy case with a documented loss of the complex I activity [50], no mtDNA deletions were detected. The S13F patient without mtDNA deletions as well as our R350P patient with mtDNA deletions both displayed a markedly reduced number of mtDNA copies. The data derived from human desminopathy patients thus give a heterogeneous picture of the mtDNA pathology. Corresponding analyses in the R349P desmin knock-in mice demonstrated the presence of very prominent mtDNA deletion species and a markedly reduced mtDNA copy number in homozygous mice. Though deletions of the mtDNA were also detected in wild-type animals, they were much more frequent in the heterozygous and homozygous conditions. Additionally, we performed analyses in age-matched desmin knock-out mice to distinguish effects due to the absence of desmin and the presence of point-mutated desmin. Though mtDNA deletions were detected in both homozygous R349P desmin knock-in and homozygous desmin knock-out mice, the former, which solely express mutant desmin at low abundance, displayed much more pronounced alterations with very prominent shortened mtDNA species and almost no full-length mtDNA. The results in our three desminopathy models, i.e., heterozygous and homozygous desmin knock-in and homozygous desmin knock-out mice, indicate that the expression of mutant desmin rather than the lack of desmin cause the mtDNA instability.

### On the way to a pathomechanism

Our own data and the literature referenced here clearly demonstrate that the expression of mutant desmin affects mitochondria on different levels in skeletal muscle tissue. The most impressive effects were noted in homozygous R349P desmin knock-in mice, in which the sole expression of mutant desmin leads to a complete disruption of the desmin intermediate filament cytoskeleton. Though this lack of a filamentous system that provides topological order may explain an aberrant subcellular distribution and consecutive damage of mitochondria, the substantial effects on mitochondrial size, abundance, distribution, enzyme activities, protein expression profiles, respiratory complex formation, and integrity of mtDNA strongly argue against a simple, concomitant secondary pathology. When translating our observations into the new concept of a mitochondrial network organization in skeletal muscle cells [20, 28] as derived by 3D-type visualization techniques, the absence of a functional desmin intermediate filament system will cause the reorganization of mitochondria into a less extended and ramified structure. The functional consequences could be a less efficient metabolite exchange between mitochondria and sarcomeric units. Moreover, since affected mtDNA integrity was noted in homozygous R349P desmin knock-in, but only to a far lesser extent in desmin knock-out mice, it is tempting to speculate that the mutant desmin exerts an additional toxic gain of function on mitochondria.

Our biochemical analysis of the homozygous knock-in genotype also revealed a consistent defect of complex I itself and within respiratory supercomplexes. As demonstrated in various neurodegenerative disorders, complex I seem to have an increased susceptibility to toxic protein species [26, 53, 70]. Since the observed mitochondrial pathology clearly preceded the clinical manifestation of muscle weakness in homozygous R349P desmin knock-in mice [16], we conclude that mutant desmin-induced mitochondrial dysfunction defines early disease stages, which significantly contribute to the progressive muscle damage in autosomal-recessive desminopathies with maintained expression of mutant desmin. When we translate this assumption into the context of the more frequently occurring autosomal-dominant desminopathies, one has to keep in mind that the toxic effects of mutant desmin on the extrasarcomeric cytoskeleton [16] and mitochondria are more focal in nature, due to the maintained expression of wild-type desmin from the non-mutated desmin allele and the observed segregation of mutant desmin from the wild-type protein [16], which leads to the coexistence of mutant desmin aggregates and a desmin filament system.

With respect to gene-targeted mice as model systems for human disease, it is of interest to mention that our previous [16] and the present study revealed marked differences between human and murine heterozygous R350P/R349P desminopathies. Human patients usually develop progressive and often devastating muscle weakness in their second to forth decade of life [15]. In contrast, our sedentary laboratory mice, although they clearly display desmin-positive protein aggregation pathology and develop a cardiomyopathy, do neither develop skeletal muscle weakness nor marked myopathological changes [16]. This difference suggests that additional factors are needed to unveil the typical disease pathology in heterozygous R349P desmin knock-in mice. Accordingly, acute and strenuous physical exercise has recently been shown to markedly accentuate the muscle fiber pathology in heterozygous W2711X filamin-C knock-in mice, which are a patient-mimicking model of filamin-C related myofibrillar myopathy [12]. Future exercise studies with the heterozygous R349P desmin knock-in mice will address the issue whether an acute energy demand accelerates the general muscle pathology and increases the degree of mitochondrial pathology. These studies are clearly needed for further counselling of patients with regard to physical exercise as well as the development of novel therapeutic strategies modulating mitochondrial dysfunction, e.g., mitochondrial biogenesis or ROS production, in desminopathies and other forms of protein aggregate myopathies.

## Electronic supplementary material

Below is the link to the electronic supplementary material.

Figure S1. Reduction of subunit proteins of all mitochondrial complexes in R349P desmin knock-in mice. Results of label-free quantitative mass spectrometric analyses of lysates prepared from total soleus muscle tissue (same pooled material derived from five mice per genotype as used in Fig. 4a) as well as laser microdissected type I muscle fibers of the lower extremities derived from wild-type (WT), heterozygous (HET) and homozygous (HOM) R349P desmin knock-in mouse littermates. The color code indicates whether a protein was found to be over- or under-represented in the heterozygous or homozygous genotype as compared to the wild-type protein level. Proteins that displayed an up-regulation of higher than 2 are marked in dark cyan, and between 1.2 to 2-fold in light cyan. A down-regulation higher than 2-fold is marked in dark magenta, and between 1.2 and 2-fold in light magenta. Proteins that were regulated less that 1.2-fold are marked in gray. The analyzed proteins were grouped by their mitochondrial and nuclear DNA origin as well as their affiliation to the different mitochondrial complexes. Proteins for which a fold change could not be calculated are marked with n.a. (not available). Unique peptides, number of measured peptides which were found to be unambiguous for this protein. Note that it is a common problem in mitochondrial proteomics that the very hydrophobic mitochondrial proteins can only be identified by few tryptic peptides. Despite of this limitation, the label-free quantification (LFQ) MaxLFQ algorithm [18] can reliably quantitate a protein based on even a single unique peptide. While the fold-changes referring to microdissected type I muscle fibers were solely calculated on the basis of unique peptides, the fold-changes referring to the soleus muscle lysates were calculated on the basis of both unique and razor peptides. Note that the desmin protein levels were markedly reduced in hetero- and homozygous animals as previously shown. Further note that proteins like GAPDH and Hsp60 usually employed as loading controls for immunoblots were also markedly regulated
