# Supplementary figures and images for: Mutant desmin substantially perturbs mitochondrial morphology, function and maintenance in skeletal muscle tissue
Source: Acta Neuropathol. 2016 Jul 8;132:453–73. doi: 10.1007/s00401-016-1592-7 (PMC4992032; doi:10.1007/s00401-016-1592-7)

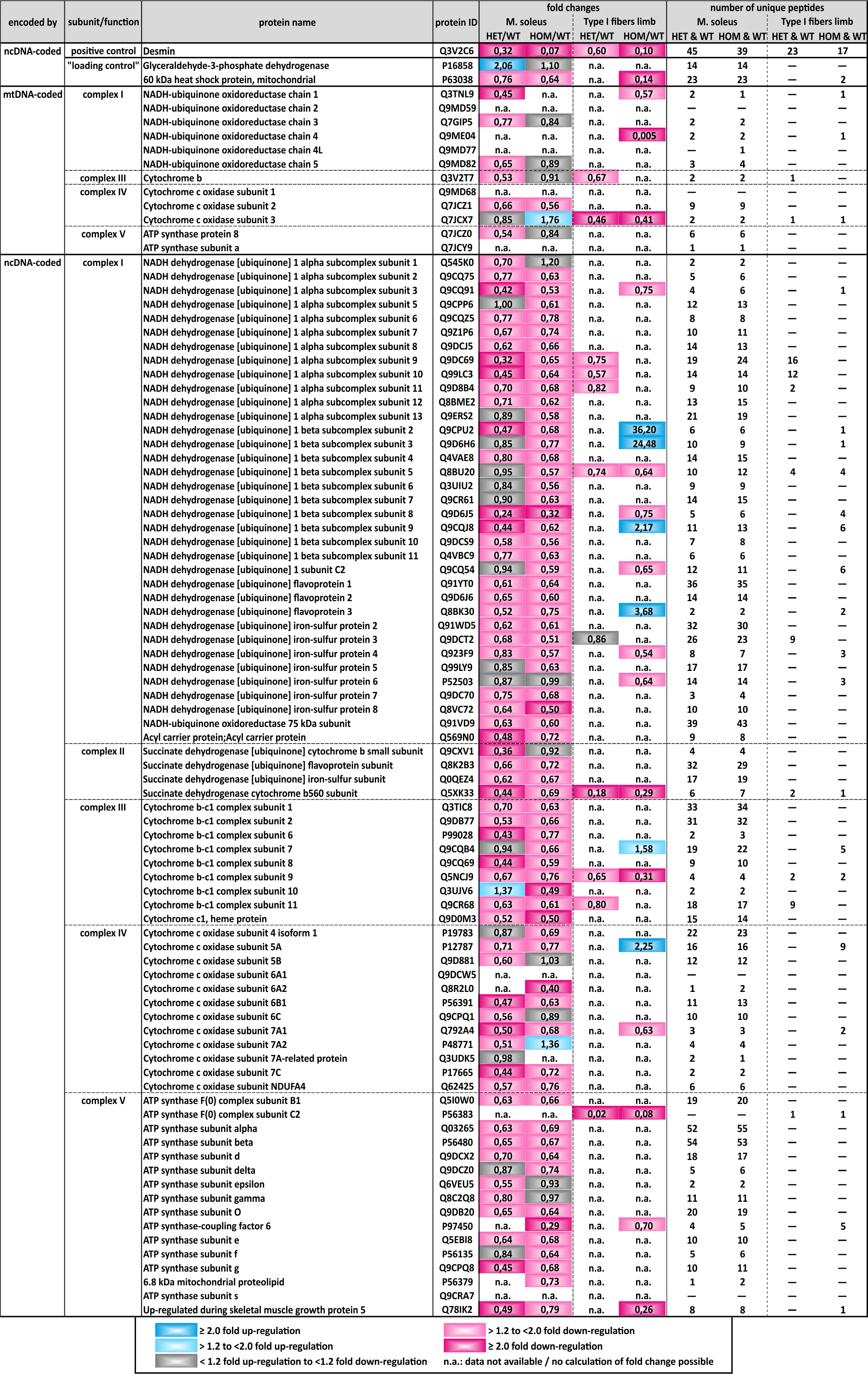

Supplement: Supplementary file 1 — Figure S1. Reduction of subunit proteins of all mitochondrial complexes in R349P desmin knock-in mice. Results of label-free quantitative mass spectrometric analyses of lysates prepared from total soleus muscle tissue (same pooled material derived from five mice per genotype as used in Fig. 4a) as well as laser microdissected type I muscle fibers of the lower extremities derived from wild-type (WT), heterozygous (HET) and homozygous (HOM) R349P desmin knock-in mouse littermates. The color code indicates whether a protein was found to be over- or under-represented in the heterozygous or homozygous genotype as compared to the wild-type protein level. Proteins that displayed an up-regulation of higher than 2 are marked in dark cyan, and between 1.2 to 2-fold in light cyan. A down-regulation higher than 2-fold is marked in dark magenta, and between 1.2 and 2-fold in light magenta. Proteins that were regulated less that 1.2-fold are marked in gray. The analyzed proteins were grouped by their mitochondrial and nuclear DNA origin as well as their affiliation to the different mitochondrial complexes. Proteins for which a fold change could not be calculated are marked with n.a. (not available). Unique peptides, number of measured peptides which were found to be unambiguous for this protein. Note that it is a common problem in mitochondrial proteomics that the very hydrophobic mitochondrial proteins can only be identified by few tryptic peptides. Despite of this limitation, the label-free quantification (LFQ) MaxLFQ algorithm [18] can reliably quantitate a protein based on even a single unique peptide. While the fold-changes referring to microdissected type I muscle fibers were solely calculated on the basis of unique peptides, the fold-changes referring to the soleus muscle lysates were calculated on the basis of both unique and razor peptides. Note that the desmin protein levels were markedly reduced in hetero- and homozygous animals as previously shown. Further note that p [file 401_2016_1592_MOESM1_ESM.tif]
